# Analytical Characterization of Pneumococcal Vaccine Conjugates Using SEC-MALS Technique

**DOI:** 10.3390/mps9020063

**Published:** 2026-04-07

**Authors:** Chloe Wright, Gowri Chellappan, Abigail Mydland, Gowthami Jagruthi Penumaka, Geetha Karengil, Harshita Seth, Anup Datta, Subhash V. Kapre

**Affiliations:** 1 Bacterial Research and Development, Inventprise, Inc., Redmond, WA 98052, USA; 2 Inventprise, Inc., Redmond, WA 98052, USA

**Keywords:** SEC-MALS, *Streptococcus pneumoniae*, conjugate vaccines, compositional analysis, rCRM197, molar mass

## Abstract

Background/Objectives: Infection from *Streptococcus pneumoniae* can lead to serious complications, such as meningitis and pneumonia, in children under 2 years of age, older adults, and immunocompromised populations. Conjugate vaccines against the pathogen have been licensed for the prevention of invasive pneumococcal disease. Conjugate vaccine development is an involved process demanding extensive characterization of both the polysaccharide (PS) and protein (Pr) moieties in complex structures. One powerful tool in our analytical tool kit that can shed light on various analytical attributes of conjugate vaccines, such as molecular weight and composition and conjugation efficiency, is the size-exclusion chromatography-multi-angle light scattering detector (SEC-MALS) technique. Herein, we demonstrate the applicability of the SEC-MALS approach for pneumococcal conjugate vaccine product characterization. Methods: Capsular polysaccharides for serotypes (STs) 1, 3, 5, 10 A, 18 C, 24 F, and 33 F conjugated to rCRM197 carrier protein were chosen for this study. Results: The technique was very straightforward, with a high degree of accuracy (>90% based on standards) and repeatability (<2% RSD) for conjugate molar mass measurements. In addition, leveraging the capability of SEC-MALS for compositional analysis, we were able to get detailed information on the molecular assembly and conformation of the conjugates and further tweak the conjugation process to yield conjugates of a desired molar mass. Conclusions: Thus, this study highlights the usefulness of the SEC-MALS technique for in-depth conjugate vaccine biophysical characterization, which is critical for achieving optimal product attributes, driving manufacturing consistency and vaccine potency.

## 1. Introduction

Pneumococcal pneumonia remains a major public health concern, especially in developing countries where vaccine accessibility is often limited. The immunocompromised and children < 2 years of age are among those with a higher risk of infection by *Streptococcus pneumoniae*, which can be fatal [1,2]. The introduction of multivalent pneumococcal conjugate vaccines has reduced the disease burden caused by this pathogen in these vulnerable populations [1]. These vaccines incorporate capsular polysaccharides from *S. pneumoniae* conjugated to a carrier protein [3]. The attachment of the carrier protein to the polysaccharide promotes a T-cell-dependent response, which is crucial for driving vaccine immunogenicity [4]. In addition, the ratio of polysaccharides to proteins impacts the immune response from a vaccine [5]. For this reason, extensive characterization of the vaccine at different stages of development is essential for ensuring product quality and purity. 

Analytical characterization is important for the identification of critical quality attributes that can have a direct impact on vaccine stability and immunogenicity. In regard to glycoconjugates, product characteristics such as conjugate molecular weight (or molar mass) and composition are some of the key metrics for process consistency and vaccine potency [6]. One of the most common methods for molecular weight determination is size exclusion chromatography-high performance liquid chromatography (SEC-HPLC). Molecules with different hydrodynamic radii elute at different times, which in turn is used for the calculation of molecular weight. Although it is a straightforward approach, one limitation of SEC-HPLC is its reliance on reference standards, which restricts the range of the calculated molar mass and does not guarantee accuracy. This method is also unable to separate molecules of the same hydrodynamic radius but differing molecular weights due to mass determination based on elution time [7]. Viscometry is another relative method of separation based on the relationship between the intrinsic viscosity of molecules and the hydrodynamic volume that is utilized for molar mass determination. A major advantage of viscometry is that it is much more affordable than other methods, but a drawback of the method is that it still relies on a hydrodynamic volume-based calibration curve for molar mass determination and is more time-consuming [8]. Further, detection of sample contaminants is difficult with viscosity-based measurements as opposed to SEC-based separations, which can help identify different species in sample chromatograms.

A technique that builds on the applications of SEC-HPLC is SEC-MALS. The light scattering detector and SEC combination system is considered to be an absolute method for molecular weight determination as it does not rely on relative standards or calibration curves, unlike techniques such as viscometry and SEC-HPLC and assumptions on the shape of the molecules [9]. Including a MALS detector is also a more reliable and pragmatic approach for determining other biophysical properties of molecules, such as size and molecular composition. In particular, what distinguishes SEC-MALS from SEC-HPLC is its ability to carry out compositional analysis of complex molecules, such as polysaccharide–protein conjugates, by leveraging the light-scattering properties of molecules in solution. In the current study, we report the analytical characterization of pneumococcal serotypes 1, 3, 5, 10 A, 18 C, 24 F, and 33 F conjugated to rCRM197 carrier protein. Several key analytical attributes of conjugates such as molar mass distribution profiles, composition, polymer conformation, and conjugation reaction kinetics were determined. We inferred the technique to be highly versatile and effective for the determination of conjugate molecular weight and PS-Pr molecular association and drug substance conjugation efficiency. Further, the applications of the technique can be extended to other complex biomolecules such as polymers, lipid nanoparticles, liposomes, and virus-like particles, etc., that require in-depth biophysical characterization as a part of quality assessment and product development [10].

## 2. Materials and Methods

### 2.1. Reagents and Materials

We purchased 10x PBS, pH 7.4, from Thermo Fisher (Waltham, MA, USA), diluted to 1x with in-house-sourced MilliQ water, and filtered twice using a 0.1 µm filter from Millipore Sigma (St. Louis, MO, USA). Bovine serum albumin (BSA) ampule (2 mg/mL) was purchased through Thermo Fisher Scientific (Waltham, MA, USA) and lot-certified by Wyatt Technologies (Santa Barabara, CA, USA). Pullulan P1300 was purchased through Agilent Technologies (Santa Clara, CA, USA) and prepared as a 1 mg/mL solution in WFI. LB-804 and LB-806 columns were installed in tandem with an LB-G guard column (Resonac America, New York, NY, USA).

### 2.2. Conjugates

Pneumococcal conjugates utilized for SEC-MALS analysis were prepared using in-house-established methods involving cyanylation chemistry with 1-Cyano-4-(dimethylamino)pyridinium tetrafluoroborate (CDAP) [11]. In the conjugation reactions involving 33 F serotype, all the input conditions, including the key process parameters such as PS molar mass (274 kDa), PS:CDAP (1:0.8, *wt*/*wt*), PS and derivatized protein concentrations (10 mg/mL, each), PS activation time period with CDAP (5 min), and PS:Pr (1:0.8, *wt*/*wt*) for batches 1 and 2 were identical, with the exception of the conjugation reaction duration being 19 h and 4 h, respectively, following protein addition. Each of the conjugates was double-filtered using a 0.22 µm Acrodisc syringe filter (25 mm) from Pall Corporation (Port Washington, NY, USA).

### 2.3. SEC Methods and Conditions

SEC was performed on a Waters Arc Premier system (Milford, MA, USA) equipped with a Sample Manager FTN-R, Quaternary Solvent Manager, integrated degasser, quaternary pump, autosampler, and a 2489 UV-Vis diode array detector. The system was run with 1X PBS, pH 7.4, as the mobile phase buffer. The flow rate was set to 0.8 mL/min, with a run time of 35 min per injection. In-line detection included a DAWN8 multi-angle light scattering detector (LS) and an Optilab refractive index (RI) detector from Wyatt Technology (Santa Barbara, CA, USA), arranged sequentially as SEC-UV-MALS-RI. The calibration of the instrument prior to its first use was done using toluene. Normalization, band broadening correction, and peak alignment to account for detector delay were performed using a monodisperse BSA standard following Wyatt user manuals. Data analysis was conducted using Astra 8 software. For molar mass measurements, curve-fitting models such as Zimm’s 1st order formalism and Berry’s 2nd order fit were compared depending on the radius of gyration (R_g_) values for the conjugates. Conjugate composition involving stoichiometric ratios of polysaccharide-to-protein and conformational analysis was calculated using the Astra protein conjugate processing method. The Astra protein conjugate method uses respective refractive index increments (dn/dc) and UV coextinction efficient values for the protein and modifier to determine the molar mass and mass fractions of the molecules [12]. The UV A_280_ extinction coefficients used were 0.934 mL/(mg·cm) for rCRM197 [3] and 0.667 mL/(mg·cm) for BSA (Wyatt Technology, Santa Barbara, CA, USA). The dn/dc values applied were 0.185 mL/g for both rCRM197 and BSA [13], 0.148 mL/g for pullulan [14], and 0.133 mL/g for pneumococcal polysaccharides based on the literature [15]. For peak analysis, baselines were selected using the auto baseline tool, followed by peak boundary selection and the application of the Astra protein conjugate processing method. Correct dn/dc and UV extinction coefficients were applied to samples, leading to the calculation of molar mass results. Samples were analyzed neat at concentrations ranging from 0.35 to 1.0 mg/mL (determined using anthrone- or uronic acid-based colorimetric assays depending on the ST), with injection volumes between 100 and 200 µL, depending on concentration.

## 3. Results

### 3.1. Conjugate Molecular Weight

#### 3.1.1. System Calibration and Verification of Analytical Performance

**Figure 1:**
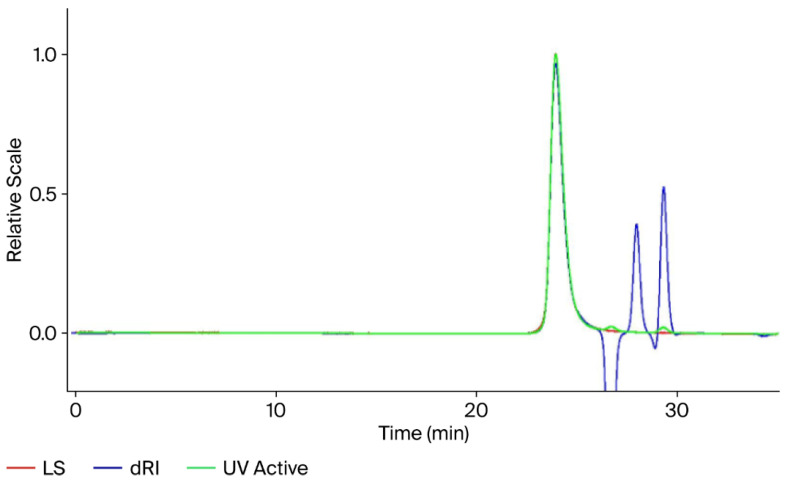
BSA chromatogram run on a SEC-UV-MALS-RI system as a function of retention time. A total of 80 µg of the protein was injected into the LB-804 and LB-806 columns in series along with 1X PBS, pH 7.0, as the mobile phase buffer at 0.8 mL/min. LS, dRI, and UV signals are represented by the red, blue, and green lines, respectively.

To ensure accuracy of the SEC-MALS technique for conjugate molar mass measurements, key aspects of instrumentation such as peak alignment, normalization of scattering intensity, band broadening correction and verification of column performance were accounted for using protein and polysaccharide polymer standards. A 40 µL injection of 2.0 mg/mL BSA and a 100 µL injection of 1.0 mg/mL pullulan p1300 were performed. A chromatogram of a BSA injection is shown in Figure 1. The weight average molecular weight (M_w_) values for BSA and pullulan p1300 were 66.4 kDa and 1220 kDa, respectively. A mass accuracy of >95% for BSA and >90% for pullulan was achieved (Table 1) when compared with values in the product CoA.

#### 3.1.2. Repeatability

**Figure 2:**
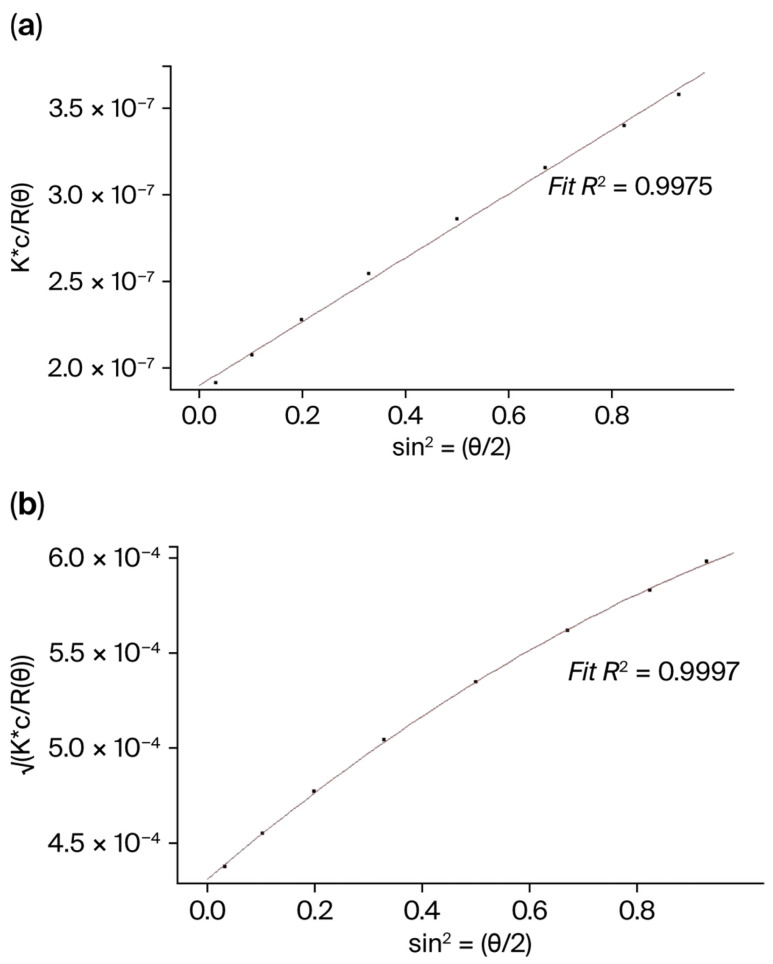
A plot of light scattering intensity vs. angular function for 24 F conjugate using (**a**) Zimm’s 1st order fit and (**b**) Berry’s 2nd order fit with R^2^ values > 0.99.

Seven pneumococcal serotypes were selected for monovalent conjugate characterization. Serotypes 1, 3, 5, 10 A, 18 C, 24 F, and 33 F were chosen based on the variations in their sugar backbone repeating unit structure, presence of different functional groups, conformation and molecular assembly with the carrier protein. For example, serotype 1 exhibits a zwitterionic behavior due to the presence of both amine and carboxylic acid groups in its structure. In the case of serotype 3, it is a linear polymer of glucose and negatively charged glucuronic acid. Serotypes 10 A, 18 C and 24 F have phosphate groups in their chemical structure. Beyond considering their physiochemical properties, conjugates encompassing a molecular weight range of 3000 to 10,000 kDa were employed for determining the column and assay performance. In the case of 33 F, which had a very high conjugate molecular weight of 23,810 kDa, the serotype was chosen to demonstrate the usefulness of the technique for in-process reaction monitoring and, in turn, for process optimization to yield a conjugate in the desired molar mass range. Each conjugate was analyzed on the MALS instrument in triplicate to ensure assay repeatability. For conjugate molecular weight determinations, the 1st order Zimm and 2nd order Berry curve fitting models were utilized, the data from which are presented in Table 2. Considering 24 F as a representative serotype, we demonstrated that satisfactory goodness of fit was achieved for both of the models, with comparable molecular weight values (Table 2) and R^2^ values > 0.99 (Figure 2). Also, the system repeatability was very high, with <2% RSD in M_w_ values among triplicate injections for each of the conjugates.

### 3.2. Compositional Analysis

**Figure 3:**
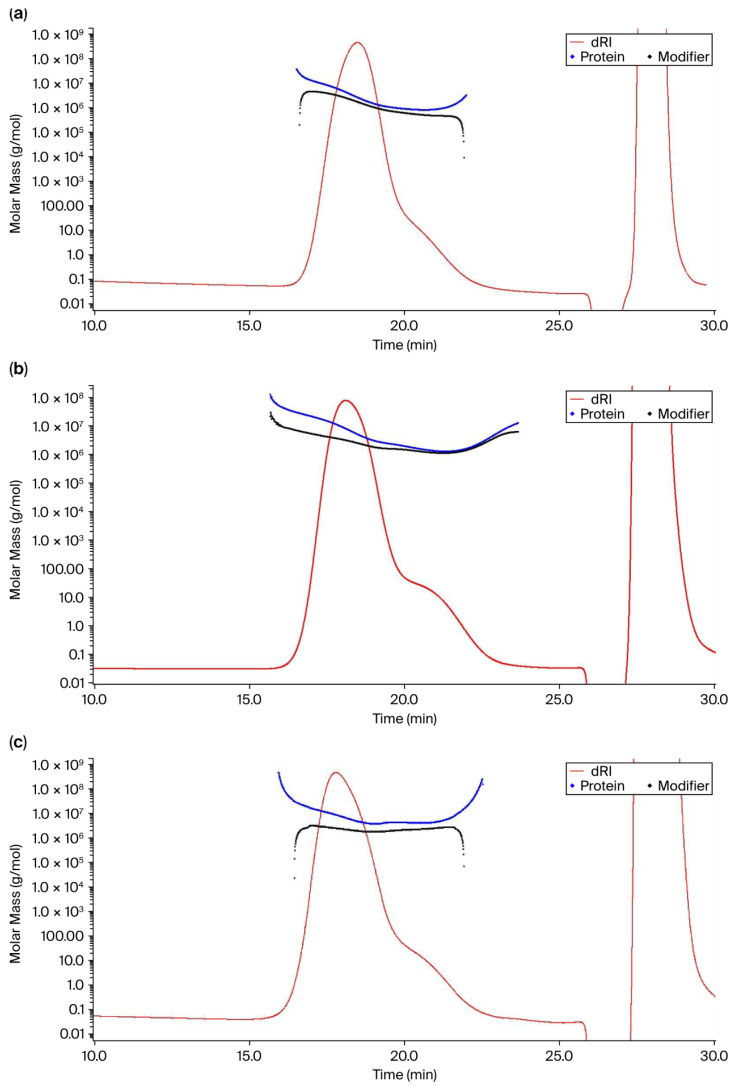
Molar mass vs. retention time graph for (**a**) ST 3 conjugate, (**b**) ST 10 A conjugate, and (**c**) ST 24 F conjugates. The modifier (polysaccharide) and protein masses are represented by the black and blue lines, respectively, while the red line represents the dRI chromatogram.

One of the significant applications of the SEC-MALS technique is the compositional analysis of molecules. Using the Astra protein conjugate analysis processing method, polysaccharide and protein concentrations for each of the conjugates were obtained. Polysaccharide-to-protein ratios (PS:Pr) calculated from the concentrations were compared with the calculated PS:Pr from polysaccharide and protein concentrations determined using colorimetric assays (Table 3). Mass recovery from the column was estimated to be 75–92% for all serotypes except for 33 F, which was around 60%. The higher protein loading compared to the PS (PS:Pr > 1:1.3) is consistent for each serotype except for PNU1 (PS:Pr = 1:1). In the conjugate molar mass vs. retention time profiles for STs 3, 10 A, and 24 F, as shown in Figure 3, it is clear from the chromatograms that the protein mass is greater than the polysaccharide mass, indicating that the protein is contributing more to the total molar mass of the conjugate. An interesting observation from the molar mass profiles in Figure 3a–c is that the elution of larger molar mass molecules occurs at a later retention time (R_t_~22–25 min), atypical in SEC-based separation. This is related to the phenomenon of pore anchoring, which occurs when highly branched molecules or molecules of very high molecular weight cannot exit the pores in an SEC column, causing them to elute later [16].

### 3.3. Conjugate Conformational Analysis

**Figure 4:**
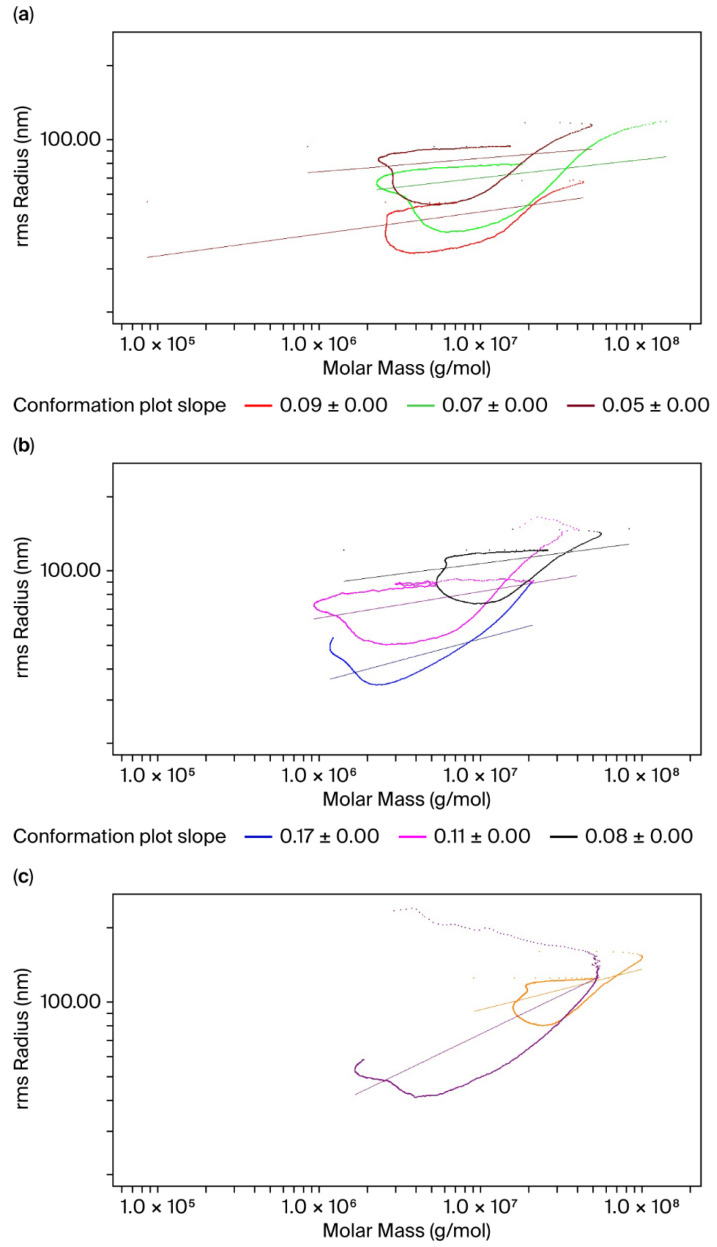
Conformation graphs for (**a**) PNU1, 10 A, 18 C (**b**) 3, 5, 24 F and (**c**) 33 F batch 1 and batch 2 conjugates represented by the red, green, brown, blue, magenta, black, orange, and purple traces, respectively.

In addition to the compositional analysis of the conjugates, we also looked for cues in the conformation of conjugates, given that each polysaccharide has a distinct sugar backbone and side chain structure and varied molecular association status with the carrier protein. A log–log plot of root-mean-square radius (RMS) vs. molar mass for the conjugates is shown in Figure 4a–c. A characteristic upswing in the conformation plot of the conjugates indicates an increase in polydispersity with an increase in molar mass, which has a more pronounced effect on the Z-average RMS (R_z_). The polymer conformation was determined using the slope (α) of the conformation plot, which was calculated to be 0.05–0.09 for ST 1, 24 F, 10 A and 18 C, 0.11 for ST 5 and 0.17 for ST 3. For the 33 F conjugates, batches 1 and 2 were prepared using different process conditions, and α values of 0.16 and 0.32 were obtained. The lower slope values compared to the typical α values of 0.4–0.6 observed for linear/random coil structures indicated that the conjugates adopted a highly branched and compact conformation.

### 3.4. Conjugation Reaction Kinetics

**Figure 5:**
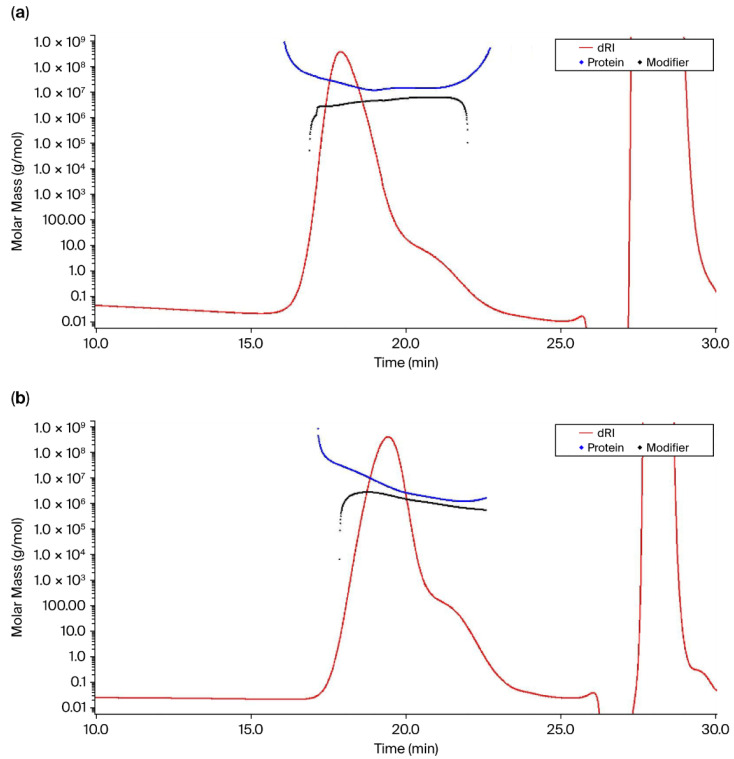
Molar mass vs. retention time graph of (**a**) batch 1 33 F conjugate and (**b**) batch 2 33 F conjugate analyzed using SEC-MALS following conjugation reaction duration of 19 h and 4 h, respectively. The protein and modifier (polysaccharide) masses are represented by blue and black lines, respectively, while the red line represents the dRI chromatogram. A characteristic hook-shaped curve was observed in the protein molar mass profile (blue trace) of batch 1 conjugate (**a**), indicating the pore-anchoring effect.

While optimizing the conjugation process, SEC-MALS was used to monitor in-process reaction kinetics. During the initial experiments with ST 33 F (hereon referred to as batch 1), a higher M_w_ value of 23,810 kDa (Figure 5a) was obtained when the conjugation reaction was carried out for 19 h. In a follow-up experiment (hereon referred to as batch 2), the reaction kinetics were monitored after 4 h, which yielded a conjugate M_w_ value of 7719 kDa (Figure 5b), which was much lower than the molecular weight of 23,810 kDa obtained from batch 1. We observed that a product with a desired molar mass around 7000 kDa was obtained for batch 2 conjugate, which utilized the same polysaccharide starting material (M_w_, 274 kDa) and conjugation input process conditions, with the only difference being the conjugation reaction duration, which was reduced to 4 h. The molar mass, protein, and polysaccharide concentration and PS:Pr (calculated from polysaccharide and protein concentrations) for batch 1 and batch 2 conjugates are shown in Table 4. Figure 5a,b represent the protein and polysaccharide molar mass vs. retention time graphs for ST 33 F batch 1 and batch 2 conjugates, respectively.

## 4. Discussion

In this study, we have utilized the SEC-MALS technique for analytical characterization of pneumococcal conjugate vaccines. Considering pneumococcal serotypes 1, 3, 5, 10 A, 18 C, 24 F, and 33 F conjugated to the rCRM197 carrier protein, physical properties of molecules such as the conjugate molar mass, molecular composition and conformation were studied. For molar mass determinations, the system’s accuracy was confirmed to be >90% with high repeatability in measurements (<2% RSD). We inferred that the conjugate molar mass determinations using Zimm’s and Berry’s formalisms (Table 2) showed good correlation in molar mass between the two methods with R^2^ > 0.99 (Figure 2 for 24 F conjugate), although Berry’s 2nd order fit is more ideal for molecules with radius of gyration (R_g_) > 50 nm and for large polymers such as polysaccharide–protein conjugates, which are the target species of interest [17].

A key aspect of the SEC-MALS technique that we explored in our study is the compositional analysis of conjugates, which allowed for the determination of polysaccharide and protein concentrations and the calculation of stoichiometric ratios of polysaccharide and protein (PS:Pr) moieties in the conjugate. SEC-MALS is unique in that aspect and advantageous because PS:Pr is a critical quality attribute for conjugate vaccine development. Since conjugate vaccines drive T-cell-mediated immune responses primarily through the carrier protein, suboptimal protein loading in the conjugate can either make the conjugate weakly immunogenic, relegating it to a polysaccharide vaccine, or suppress the immune responses to the carbohydrate antigen, likely through the phenomenon of carrier-mediated immune suppression [18]. We observed lower PS:Pr for all conjugates except for ST 1 (PS:Pr = 1:1), as shown in Table 3. However, PS:Pr determined using colorimetric assays indicated the PS:Pr to be 1:0.4–1:1.8 (Table 3). The reasons for the unexpectedly low concentrations of PS obtained from the MALS system could be multi-fold. Firstly, the average dn/dc value of 0.133 mL/g that was applied for the PS samples might not be universally applicable to all serotypes since values in the literature suggest slightly different dn/dc values (0.130–0.139) based on the ST [19]. To address this gap, we are currently empirically determining the dn/dc of each serotype polysaccharide using the SEC-optilab RI detector system, which will be used for all molar mass measurements. Secondly, 100% mass recovery might not be achievable for samples, likely due to sample adsorption to the column, which can in turn lead to inaccurate PS concentration determination and skew the PS:Pr to lower values. In this regard, exploration of novel stationary phases such as ethylene-bridged hybrid (BEH) columns with wide particle pore sizes can be beneficial for efficient separation of large molecular weight conjugate polymers. Thirdly, to a minor extent, it could be related to the lower S/N from the RI detector arising from lower amounts of sample injection (possibly due to errors in PS concentration determined from colorimetric assay), although distinct RI peaks were observed in the molar mass profiles of the conjugates under discussion.

Another interesting observation from the compositional analysis of conjugates is related to the pore anchoring phenomenon, based on the shape of the conjugate molar mass curves. In an ideal SEC separation, molar mass decreases with elution time as smaller particles elute after larger particles. Pore anchoring occurs in SEC columns involving branched polymers or molecules with high molar mass [16]. The shape of the branched polymers prevents the molecules from exiting the column pores after they have entered, thereby causing them to elute later. As shown in Figure 3 and Figure 5, the conjugate chromatogram showed that the molar mass profile exhibited a “hook” shape, or an increase in molar mass towards the end of the elution time, leading to a non-ideal SEC behavior. To further explore the pore-anchoring effect caused by branched polymers, we looked at the conformation plot of Z-average RMS radius (R_z_) as a function of molar mass, where the slope (α) of the plot can reveal details of polymer branching. The shape of the curve with upswings in R_z_ values was a result of an increase in polydispersity with an increase in molar mass, reflecting a highly heterogenous polymeric conjugate (Figure 4). To provide a simplified and qualitative assessment of the conjugate conformation, the scaling exponent trends that were derived from the SEC-MALS data indicated a highly branched conjugate molecule adopting a compact structure like sphere [20]. An in-depth analysis of the conjugate conformation could not be carried out since the SEC-MALS method fails for large and extensively branched molecules such as conjugates and does not generate reliable conformation plots [16]. The non-ideal SEC behavior arising from pore-anchoring, analyte–column interactions, and other factors can mask true information on polymer conformation and the actual branching nature. In addition to the pore-anchoring effect, the compositional analysis also revealed that the protein molar mass was higher than the polysaccharide molar mass for most of the serotypes. This observation is indicative of suboptimal protein loading on the sugars in the glycoconjugate. To address the above issues, we considered 33 F for process optimization given the unimaginably high M_w_ values (23,810 kDa, Table 4) obtained for that conjugate. Again, we employed SEC-MALS to monitor the conjugation reaction kinetics. After reducing the reaction time duration to 4 h for batch 2, a conjugate of lower M_w_ (7719 kDa) was obtained (Table 4). Further, the compositional analysis of the ST 33 F batch 2 conjugate revealed that the hook shape in the protein molar mass profile was much less pronounced (Figure 5b), suggesting that the pore-anchoring effect was minimized in comparison to the batch 1 conjugate (Figure 5a). Again, without drawing any major conclusions from the plot, a generalized, qualitative finding was that, with the changes in the conjugation process conditions for 33 F, the formation of an extremely branched and very high molecular weight polymer was prevented and a conjugate of desirable molar mass (7719 kDa) was obtained. However, the modifications in the process conditions used in batch 2 did not result in any changes to PS:Pr and it was similar to that of the batch 1 conjugate (PS:Pr—1:2.4 and 1:2.5 for batch 2 and batch 1, respectively (Table 4)). As a part of improving the conjugation process, we will modify input process conditions and apply the SEC-MALS technique as a tool to monitor conjugation reaction kinetics for achieving an ideal PS:Pr ratio of 0.7–1.3, consistent with the in-house established target range. Thus, the in-depth characterization that the SEC-MALS system allows, highlights the power of the technique in the development of complex molecules such as conjugate vaccines, with quality built into the product throughout development.

While SEC-MALS is incredibly useful for detailed analytical characterization of biomolecules, there are some limitations with the technique, and in this study, that has to be acknowledged. Although the system’s accuracy and repeatability were tested as preliminary parameters in the study, other aspects of method qualification such as linearity and robustness were not available, although they are currently being evaluated. Specifically, the concentration-dependent effects on molar mass arising from non-specific solute–solvent interactions in conjugate molecules can preclude accurate M_w_ determinations. Hence, a linearity test with the inclusion of a correction factor to minimize concentration-dependent changes to M_w_ is warranted. A second limitation is related to the compositional analysis of molecules using the protein conjugate method on Astra. The polysaccharide–protein conjugates that were made in-house included a third modifier, a hydrazide-polyethylene glycol-hydrazide (Hz-PEG-Hz, 1 kDa) linker, which was derivatized to the protein before conjugation to the polysaccharide. With the mass fraction of the linker accounting for <5% of the total mass of the conjugate, quantification of the linker in the conjugate was not feasible. As with the drawbacks of the pore anchoring effect, we plan on testing SEC columns of different stationary phase pore and particle sizes that can handle substantially higher back pressures and provide good resolution. For example, novel, wide-pore (1000 Å and 2000 Å) GTxResolve premier series SEC columns employing advanced particle phase technologies such as the bridged ethylene HO-PEO (polyethylene oxide) stationary phase have been shown to minimize non-ideal SEC behavior and provide true separation of highly heterogeneous polysaccharide–protein conjugates (unpublished information). Alternatively, AF4 is a more promising technique based on the Brownian motion principle that has shown promise in eliminating the pore anchoring effect, since it can handle M_w_ measurements of larger molecules and is free of stationary-phase interactions [21]. Further, the conformation plots exhibit a linear behavior and can reveal the true nature of polymer branching [16].

## 5. Conclusions

Polysaccharide–protein conjugate vaccines are biologics existing in complex conformational and polymeric states. In the current study involving the analytical characterization of pneumococcal conjugates, we utilized the SEC-MALS technique to determine product quality attributes such as conjugate molar mass, polydispersity, and protein and polysaccharide concentrations. The molar mass accuracy and method repeatability were established using pneumococcal conjugates differing in their monosaccharide repeating unit structure and chemical nature. In addition, we were able to decipher information on individual conjugate conformation and composition. Compositional analysis yielded great insights into the molecular assembly of each serotype and the level of protein loading on glycans in the heterogeneous product. Utilizing the technique for in-process conjugation reaction monitoring of ST 33 F, combined with compositional analysis, helped us achieve optimal conjugation efficiency and obtain a conjugate in the desired molar mass range. We are continuing to harness the in-depth characterization capabilities of this technique for conjugation process optimization tailored to each serotype and generation of glycoconjugates with optimal polysaccharide–protein ratios. In effect, key information garnered on the physical properties of glycoconjugates can play a critical role in the development of an effective quality control strategy that defines vaccine quality and immunogenicity.

## Figures and Tables

**Table 1:** Molar mass and mass accuracy of BSA and pullulan p1300 standards determined using SEC-MALS system.

Replicate	Sample	Molar Mass (M_w_, kDa)	Mass Accuracy (%)
1	BSA	66.1	99.55
2	BSA	66.1	99.55
3	BSA	66.2	99.70
1	p1300	1134	92.38
2	p1300	1129	91.97
3	p1300	1131	92.09

**Table 2:** Weight average molecular weight (M_w_) and % RSD among triplicate sample injections for pneumococcal conjugates determined using Zimm’s and Berry’s formalisms.

Serotype	Berry’s 2nd Order Fit		Zimm’s 1st Order Fit	
	M_w_ (kDa *)	% RSD	M_w_ (kDa *)	% RSD
1	6962	1.28	6929	1.16
	6897		7090	
	6788		6995	
3	5174	1.06	5222	1.42
	5164		5218	
	5075		5349	
5	3409	0.49	3385	1.16
	3429		3448	
	3396		3375	
10 A	9289	0.04	9339	1.17
	9294		9526	
	9288		9534	
18 C	6308	0.37	6302	0.74
	6280		6328	
	6281		6237	
24 F	10,297	1.77	9989	1.20
	10,196		10,209	
	9948		10,187	
33 F	24,116	0.48	24,209	0.67
	24,144		23,919	
	23,930		24,191	

* M_w_ values from Astra 8 software were rounded to the nearest whole number.

**Table 3:** Polysaccharide and protein concentrations and PS:Pr for pneumococcal polysaccharide-protein conjugates determined using SEC-MALS technique and colorimetric assays.

Serotype	Injected vol. (µL)	Mass Recovery (%)	PS Conc. by MALS (mg/mL)	Pr Conc. by MALS (mg/mL)	PS:Pr (MALS)	PS:Pr (Colorimetric Assay)
1	100	92.2	0.5	0.5	1:1.0	1:0.6
3	100	87.7	0.7	1.1	1:1.6	1:1.1
5	100	89.2	0.2	0.4	1:2.0	1:1.3
10 A	100	75.2	0.6	1.2	1:2.0	1:1.0
18 C	100	75.6	0.4	0.6	1:1.5	1:0.8
24 F	100	82.1	0.2	0.5	1:2.5	1:1.4
33 F	100	60.0	0.2	0.5	1:2.5	1:1.5

Data values reported for PS and Pr concentrations and PS:Pr were rounded to the nearest tenth.

**Table 4:** Compositional analysis of ST 33 F conjugate batches 1 and 2 determined using SEC-MALS technique.

Batch	Injected Mass (µg)	Molar Mass (M_w_, kDa)	PS Conc. (mg/mL)	Pr Conc. (mg/mL)	PS:Pr (MALS)	PS:Pr (Colorimetric)
1	80	23,810	0.2	0.5	1:2.5	1:1.5
2	80	7719	0.8	1.9	1:2.4	1:1.4

Data values reported for PS and Pr concentrations and PS:Pr were rounded to the nearest tenth.

## Data Availability

The data presented in this study is available in this article.

## Abbreviations

The following abbreviations are used in this manuscript:

LS	Light scattering
CDAP	1-Cyano-4-(dimethylamino)pyridinium tetrafluoroborate
PS	Polysaccharide
SEC-MALS	Size exclusion chromatography-multi-angle light scattering
RSD	Relative standard deviation
RI	Refractive index
ST(s)	Serotype(s)
Pr	Protein
RMS	Root-mean-square radius
